# Left ventricle strain and T1 mapping evaluation in a mouse model with myocardial infarction

**DOI:** 10.1038/s41598-025-09699-0

**Published:** 2025-08-12

**Authors:** Khaoula Bouazizi, Thulaciga Yoganathan, Frank Kober, Gwennhael Autret, Perrine Marsac, Clément Delacroix, Shannon Soulez, Mohamed Zarai, Vincent Nguyen, Adil Squalli, Paul Alayrac, Estelle Robidel, Bertrand Tavitian, Alban Redheuil, Jean-Sébastien Hulot, Nadjia Kachenoura

**Affiliations:** 1 Laboratoire d’Imagerie Biomédicale (LIB), Sorbonne Université, INSERM, CNRS, Paris, France; 2 ICAN Imaging, Institute of Cardiometabolism and Nutrition (ICAN), Paris, France; 3 Université Paris Cité, INSERM, PARCC, Paris, F-75015 France; 4 Center for Magnetic Resonance in Biology and Medicine (CRMBM), CNRS, Marseille, France; 5 Unité d’Imagerie Cardiovasculaire et Thoracique (ICT), Pitié-Salpêtrière Hospital AP-HP, Sorbonne Université, Paris, France

**Keywords:** Cardiac MRI, Mice, Strain, T1 mapping, Myocardial infarction., Biomarkers, Cardiovascular diseases

## Abstract

Myocardial strain and T1 mapping offer precise evaluation of cardiac function and tissue characteristics. The aim of this study is to provide normative myocardial strain and T1 values in mice model along with their changes after myocardial infarction (MI) using a tailored acquisition and analysis protocol. Healthy mice had MRI before and after MI. Imaging was performed to assess cardiac function and left ventricle (LV) strain. A Look-Locker Inversion-Recovery sequence was used for T1 maps reconstruction. Gadolinium doses and timing of image acquisitions were optimized. Radial and circumferential strain measurements were conducted using a custom software based on feature tracking. To address ECG signal interference, adjustments were made for accurate strain calculations. An LV dilation in MI compared to wild-type (WT) with a decrease in LV ejection fraction (*p* < 0.0001) were reported while stroke volume and cardiac output remained preserved (*p* > 0.21). Normative strain and T1 mapping data were provided revealing an increase in circumferential strain from base to apex (basal: –14 ± 1%, mid: –15 ± 2%, apical: – 21 ± 3%; *p* < 0.0001) and notable differences in native and post-contrast T1 values between mid-ventricular and apical LV segments. Circumferential strain differences between MI and WT mice were pronounced, with a significant drop in apical strain (*p* < 0.0001) in MI. Post-MI mice exhibited higher native T1 and lower post-contrast T1 compared to WT. Our methodological approach not only refines imaging sequences but also offers insights into cardiac function and tissue characteristics in mice. By describing normative values of strain and T1, our findings lay the groundwork for future research, particularly in drug therapy.

## Introduction

Myocardial strain assessment through magnetic resonance imaging (MRI) has evolved as a robust and versatile method for the comprehensive evaluation of cardiac function^1^. Using techniques such as cine displacement encoding with stimulated echoes (DENSE)^2^, strain-encoding (SENC)^3^ and feature tracking (FT)^4,5^ regional and global myocardial deformation can be accurately quantified with high spatial and temporal resolution. While DENSE and SENC are promising and evolving sequences not yet validated in large cohorts, FT, which is applied to the widely available conventional cine MRI images, has proven of utmost usefulness in studying heart chambers mechanics in large groups of patients with various cardiovascular diseases, including myocardial infarction, heart failure^6^, and hypertrophy^7^.

T1 mapping is a valuable imaging technique for the evaluation of myocardial involvement in various cardiac and non-cardiac diseases and conditions. It offers a detailed insight into myocardial tissue characteristics^8^ as it measures local quantitative T1 values, which vary according to tissue composition, extracellular space expansion^9^, fat content^10^ and infiltration such as amyloidosis^11,12^ and free water accumulation seen in myocardial edema^13^ and myocarditis^14^. When used before and after contrast agent injection, T1 mapping accordingly allows quantitative assessment of myocardial fibrosis and inflammation^15^.

The widespread availability of MRI data in large cohorts of patients with diverse and complex cardiomyopathies has revolutionized our understanding of both myocardial strain and myocardial tissue characteristics, positioning such evaluations as an integral part of routine clinical care^16,17^. Myocardial strain, encompassing longitudinal, radial and circumferential dimensions, has provided important insight into cardiac mechanics^18,19^. T1 mapping provides crucial understanding of various cardiomyopathies, helping in the differential diagnosis and treatment planning^20–23^. However, when it comes to studying similar parameters in murine models, a stark contrast emerges because of the scarcity of data and the complexity of imaging small and highly mobile structures. While the translation from human to mouse models presents difficulties^24,25^, the ability to explore myocardial functional and structural involvement in murine studies holds the potential to unravel novel insights and bridge gaps in our understanding of cardiac pathological pathways in dedicated models.

Accordingly, our study aims to provide a comprehensive evaluation of both myocardial strain and T1 mapping in a murine model before and after myocardial infarction (MI) using a tailored acquisition and analysis protocol. The implemented protocol incorporates advanced techniques to optimize the acquisition and image processing parameters, ensuring superior image quality and accurate depiction of “normal” myocardial strain dynamics as well as T1 relaxation times along with their alteration post-MI. A mouse model of MI was chosen since MI stands as a significant contributor to cardiovascular morbidity and mortality, necessitating a thorough exploration of the multifaceted changes occurring in the myocardium^26^. Also, because it is a well-established model of regional remodeling and defect in contractility and myocardial tissue characteristics.

## Materials and methods

### Animals

Experiments were approved by the Animal Ethics Committee of the French Ministry of Research (agreement number 19064/21040). All experiments were performed in accordance with relevant guidelines and regulations. Healthy male C57BL6/J mice were enrolled into our study from the animal facility of PARCC (Paris Cardiac Research Center, Paris, France). Animals had free access to food and water and were housed with a light to dark cycle of 12 h. At the end of experiments, animals were sacrificed by intraperitoneal injection of euthanasol. This study is reported in accordance with ARRIVE guidelines.

### Animal model of myocardial infarction

**Fig. 1:**
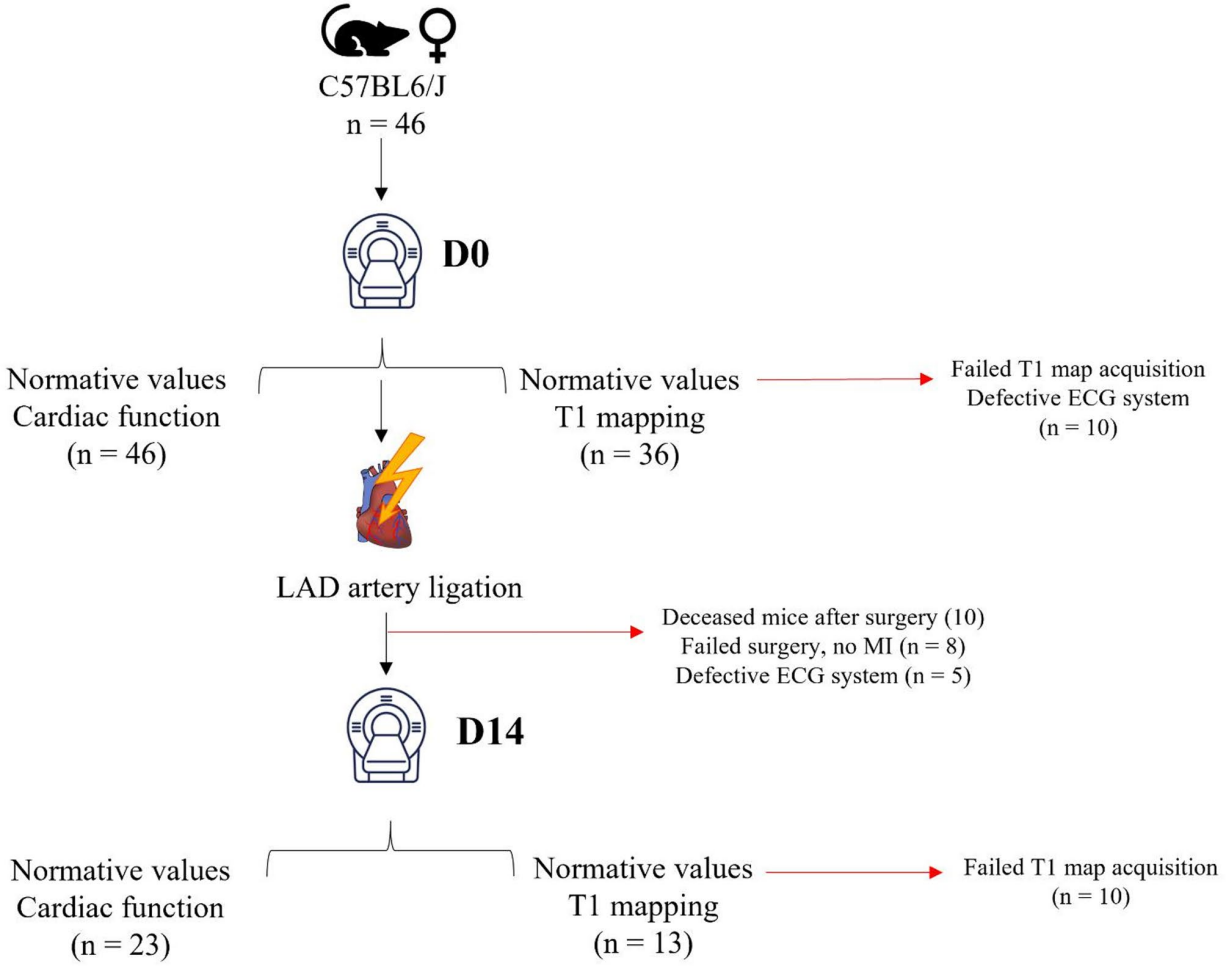
Flow chart of the study.

The surgical induction of the myocardial infarction in 10-weeks-old male C57BL6/J mice consisted of a thoracotomy under endotracheal intubation followed by permanent ligation of the left anterior descending (LAD) artery of the beating myocardium. The thoracotomy incision was sutured while removing air from the chest. The animals were monitored during the hours following the surgery up to 14 days post-MI. MRI acquisitions were performed before the surgery and 14 days after MI induction in mice that survived the surgery. A study flowchart is detailed in Fig. 1.

### Cardiac MRI acquisitions

MRI acquisitions were performed in a preclinical 4.7 T MRI system (Bruker BioSpec 47/40 USR, Ettlingen, Germany) with a 7 cm inner diameter resonator for emission and a phase array surface coil for reception that was specific to the mouse. During acquisitions, animals were heated to maintain their body temperature at 37 °C and were under isoflurane anesthesia which has the advantage of short induction and fast recovery times. The animals’ respiration rate, around 70 breaths/min, heart rate and rectal temperature were monitored throughout the MRI imaging session. After initial localization images (scout imaging on three different orthogonal planes), ECG-gated experiments were conducted by applying a Fast Low Angle Shot gradient echo (FLASH) cine sequence covering the left ventricle (LV) (6 to 7 slices) using the following average scan parameters: TR = 7.2 ms; TE = 2.7 ms; flip angle = 18°; field of view (FOV) = 30 × 30 mm^2^; matrix size = 256 × 256; planar resolution = 117 μm; slice thickness = 1 mm; number of frames = 16 per cardiac cycle, which was the best compromise given the available TR and an acceptable spatial resolution and SNR. A sufficient number of dummy scans (lasting approximately 1 s) were run to bring the magnetization to steady state prior to the imaging data readout. Total scan time was in average 2 min 18 s for a single slice.

The cine intra-gate sequence data was reordered and reconstructed retrospectively with averaged heart and respiration rate values. Acquisitions were performed using Paravision software 6.0.1 (Bruker, Ettlingen, Germany).

An ECG-gated Look-Locker Inversion-Recovery gradient-echo sequence^27^ was used to acquire 40 images at different inversion times (TI) on two short-axis slices (mid-LV and apical) with the following scan parameters: TE = 2.21 ms, minimum TR = 80 ms, flip angle = 15°, pixel size = 0.19 × 0.39 mm^2^, pixel bandwidth = 390 Hz/pixel, NEX = 1. The actual TR (the TI spacing) was equal to the individual RR-interval. T1 maps were then reconstructed using an off-line software. Post-contrast T1 images were acquired 15 min after intravenous injection of 0.8 mMol/kg of contrast enhancement agent (DOTAREM^®^, Guerbet, France).

The gadolinium injection dose and time of onset of acquisitions were optimized before this study, and testing was conducted with doses mirroring those used in human studies and then gradually increased to comply with the mice high heat-rate and the subsequent rapid wash-out: one dose, two doses, four doses, and five doses. The results indicated that the optimal dose for mice corresponds to the four-dose regimen which was equivalent to 0.8 mMol/kg.

### Image analysis

End-diastolic (ED) and end-systolic (ES) LV masses and volumes were obtained by tracing the LV endocardial and epicardial borders on the stack of short-axis FLASH images at the end-diastolic and end-systolic phases using the QMass software v4.0 (Medis, Leiden, The Netherlands).

**Fig. 2:**
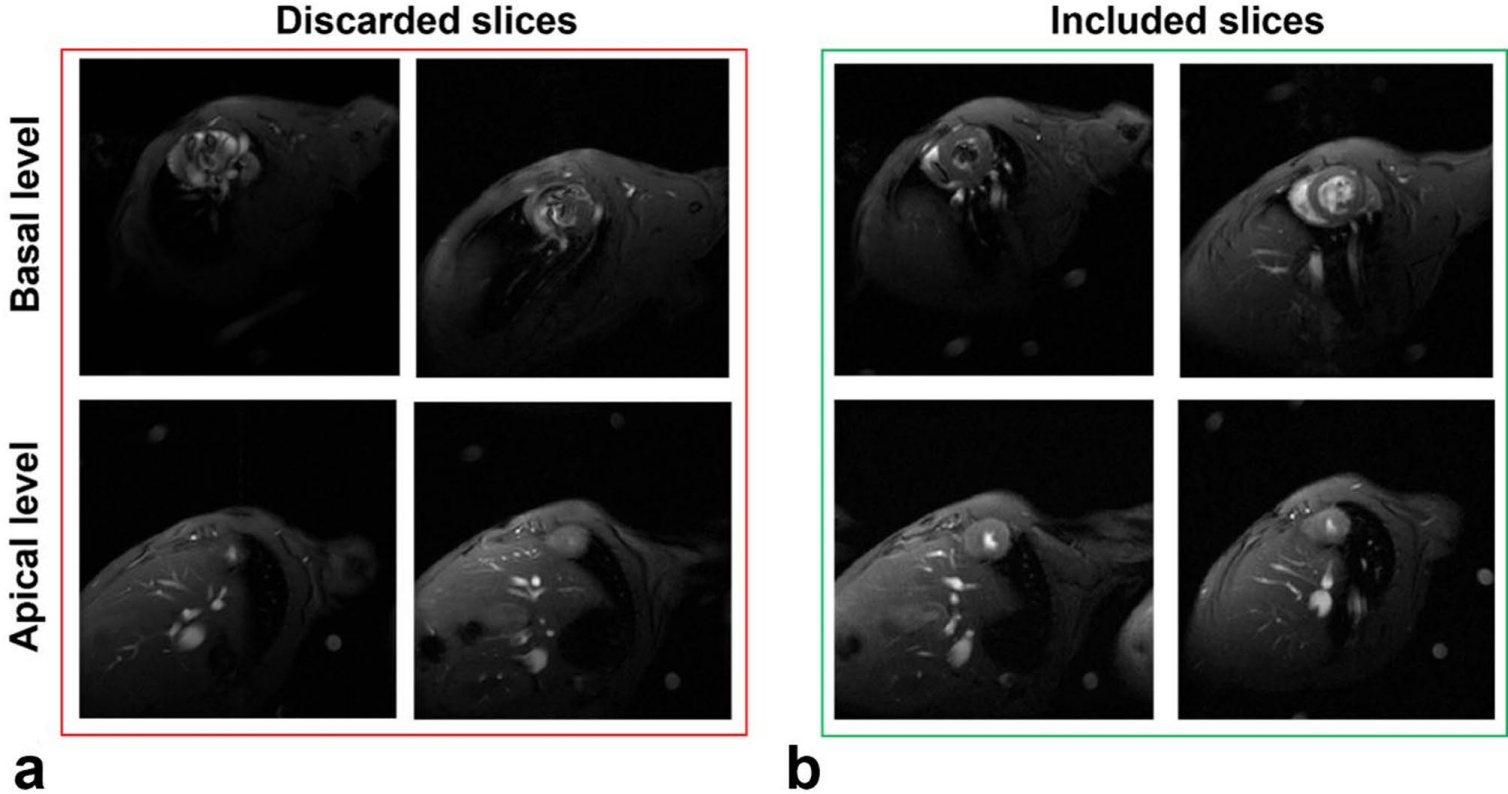
Examples of discarded (**a**) and included (**b**) slices from the tracking analysis. Basal slices were discarded because of out-flow tract and apical slices were discarded when the cavity disappears at systolic phase.

Strain measurements were performed on cine sequences using a semi-automated custom software, CardioTrack (LIB, Sorbonne University, Paris, France), based on a feature tracking algorithm^28^, previously validated against histological measures of fibrosis and fat in human subjects undergoing surgery^29^, in aging^30^ and in obese patients with diabetes^31^ as well as in a rat model of Takostsubo^32^. Basal slices contaminated with out-flow tract artifacts as well as apical slices contaminated with partial volume effects were discarded from strain analysis. Basically, a slice is included in strain analysis only if the cavity is seen within a closed circular myocardial shape throughout the cardiac cycle (Fig. 2).

**Fig. 3:**
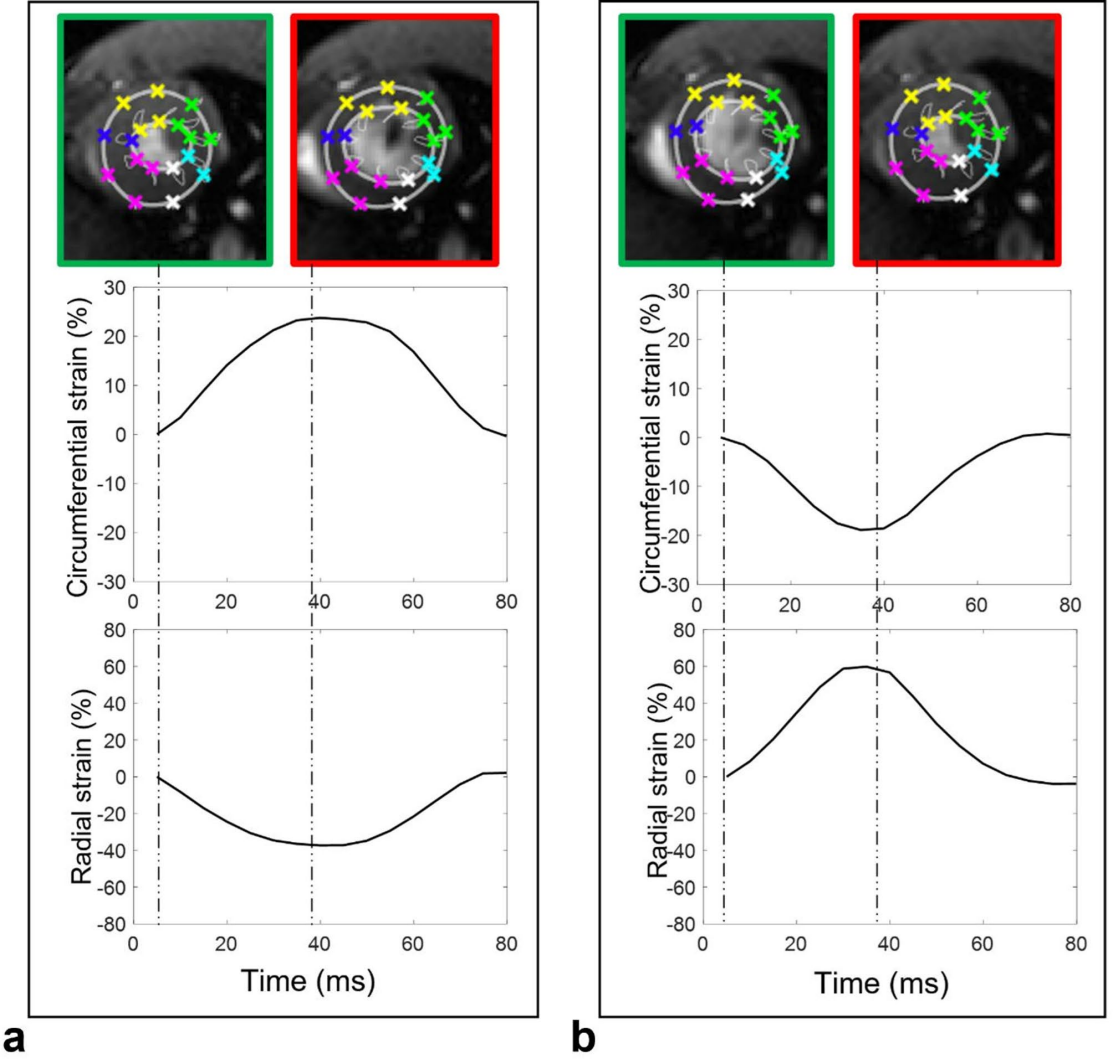
Radial and circumferential strain curves before (**a**) and after (**b**) cardiac cycle phase shift. End diastolic phase is highlighted in green while end systolic phase is highlighted in green for both panels. Panel a illustrates an erroneous positive circumferential strain and negative radial thickening for falsely triggered images and Panel b illustrates the consistency of strain measures when the cardiac cycle phases were shifted to start at end-diastolic phase.

Initial contours down-sampling was employed in mice images to accommodate for anatomical differences compared to images obtained in humans. End-systolic phase was detected and LV endocardial and epicardial borders were manually initialized on a single time phase corresponding to the LV maximal dilation. These initial contours were then automatically tracked throughout the cardiac cycle^28^ and resulting borders were used to extract time-varying strain curves of the myocardium. This was achieved by computing the temporal variations in radial myocardial thickness and circumferential myocardial length, relative to and normalized by their initial dimensions measured at the onset of the cardiac cycle. Strain-rate time varying curves were calculated as the time derivatives of strain curves. Of note, radiofrequency pulses and gradient switching often induced interferences in the ECG signal and caused false triggers. This issue, not seen in human acquisitions, was corrected in the proposed software by a circular shift of the cardiac cycle while setting its starting phase to the beginning of diastole. While this shift can be ignored for volumetric measures, as the operator can visually pick-up the end-systolic and end-diastolic phases, it plays a major role for strain calculation since the dimensions of the LV during the beginning of diastole are needed for calculating the relative deformation of the myocardium, as described above. Consequences of such false triggers and their corrections are illustrated in Fig. 3.

Peak strains and strain rates were automatically depicted from time-resolved strain and strain rate curves. Global as well as regional circumferential (CS) and radial (RS) peak systolic strain were derived from short-axis views, respectively. Peak systolic as well as early diastolic and late diastolic peak strain rate were also estimated. When these peaks were not correctly detected, the user could adjust them to the correct position. Of note, such strain quantitative measures were derived on a slice basis (apical, mid-LV and basal slices) as well as on a global basis, by averaging the three slice measures. Longitudinal strain measurement was technically feasible, however long-axis acquisitions were only done in a subset of animals.

T1 maps were reconstructed using an offline pixel-by-pixel fitting software, while considering acquisition-specific TI values. Global native and post-contrast T1 were measured from two reconstructed T1 maps (mid-LV and apical level) after endocardial and epicardial contouring. Using the anterior left/right ventricular insertion point as a reference, T1 maps were segmented according to the American Heart Association to obtain regional T1 values. The maximal native T1 and minimal post-contrast T1 were estimated over all segments and were respectively called: nat_T1_max_, post_T1_min_. Maps with significant noise attributed to poor ECG signals were excluded from the analysis.

### Histological analysis

**Fig. 4:**
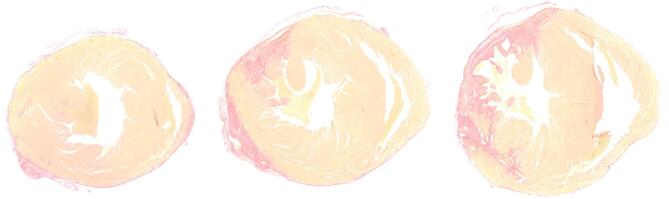
Representative section of Sirius red staining in the LV apex and base.

The LV was carefully dissected, then fixed in 4% formaldehyde for 24 h at 4 °C. After fixation, tissues were transferred to 70% ethanol for preservation before being embedded in paraffin. For histological evaluation, hearts were sectioned at 6 μm thickness using a cryostat (CM3050 S, Leica, Germany). Sections were collected from five predefined levels of the apex (6 sections per level, spaced 100 μm apart), and three levels in the basal region (6 sections per level, spaced 200 μm apart), ensuring comprehensive sampling of the entire LV wall (Fig. 4). Histological analyses were performed in a subset of animals (*n* = 5). Fibrosis was assessed using Sirius red, which allows clear visualization and quantification of collagen deposition in myocardial tissue. Images were acquired using a high-resolution slide scanner, and quantitative fibrosis analysis was performed with fibrotic area expressed as a percentage of the total myocardial area.

### Statistical analysis

Quantitative continuous variables are presented as mean ± standard deviation. Strain measurements from the three levels (base, mid-LV and apex) were compared using one-way repeated analysis of variance (ANOVA). For comparisons between WT and MI mice, a parametric Student t-test was performed. All statistical analyses were performed using Prism, version 9 (GraphPad Software; San Diego, CA, USA). Differences were compared using a one-way analysis of variance. Statistical significance was set at *p* < 0.05 (* *p* < 0.05, ** *p* < 0.01, *** *p* < 0.001). We based our statistical power analysis on a previous study in which we conducted scan-rescan measurements. This study^28^ showed that to detect a 10% difference in strain, a sample size of 11 subjects was required to reliably characterize such a difference.

## Results

MI and WT mice basic characteristics are detailed in Table 1. One might note that the success rate of strain measurements was higher than that of the T1 measurements, as only 36/46 WT and 13/23 MI mice provided a sufficiently stable ECG for the T1 acquisitions due to a deficient ECG system which was replaced during the study. Heart rates and body weights were equivalent between WT and MI groups. LV volumetric measures highlighted a significant LV dilation in the MI mice as compared to WT. This dilation came with a significant drop in LV ejection fraction (EF) in MIs as compared to WT mice, while stroke volume and cardiac output were preserved (Table 1) at the expense of LV remodeling.

**Table 1:** Main characteristics of the study population before and after myocardial infarction. Bpm: beats per minute, CI: confidence interval, CO: cardiac output, ED LVM: end-diastolic left ventricle mass, EF: ejection fraction, HR: heart rate, LV ESV/EDV: left ventricle end-systolic/end-diastolic volume, MI: myocardial infarction, SV: stroke volume.

	WT	MI	*p*-value	CI (lower to upper)
n (strain/T1)	46/36	23/13		
Weight (g)	26.4 ± 3.5	26.6 ± 3.0	0.8012	−1.649 to 2.127
HR (bpm)	508.2 ± 58.4	500.3 ± 65.0	0.6020	−38.01 to 22.19
ED LVM (mg)	53.3 ± 13.4	65.9 ± 30.5	0.0193	2.090 to 22.94
LV EDV (µl)	47.9 ± 7.4	75.78 ± 35.0	< 0.0001	17.17 to 38.53
LV ESV (µl)	18.4 ± 5.7	48.02 ± 37.0	< 0.0001	18.52 to 40.65
EF (%)	62.1 ± 9.9	42.5 ± 16.7	< 0.0001	−25.93 to −13.27
SV (µl)	29.4 ± 6.6	28.0 ± 9.2	0.4403	−5.288 to 2.326
CO (ml/min)	14.8 ± 3.3	13.7 ± 4.0	0.2135	−2.892 to 0.6580
Circumferential peak systolic strain (%)	−15.5 ± 2.8	−9.9 ± 4.0	< 0.0001	3.900 to 7.305
Radial peak systolic strain (%)	50.2 ± 10.0	34.5 ± 13.2	< 0.0001	−21.47 to −9.903
Circumferential strain rate at systolic phase (s^−1^)	−7.8 ± 0.8	−4.5 ± 1.9	< 0.0001	2.692 to 4.027
Radial strain rate at systolic phase (s^−1^)	25.2 ± 4.4	16.4 ± 6.6	< 0.0001	−11.50 to −6.034
Native global T1 (ms)	1615 ± 146	1686 ± 164	0.1518	−27.16 to 169.8
Native max T1 (ms)	1675 ± 149	1787 ± 187	0.0359	7.692 to 216.3
Post-contrast global T1 (ms)	1072 ± 52	978 ± 59	< 0.0001	−133.5 to −55.58
Post-contrast min T1 (ms)	996 ± 61	858 ± 99	< 0.0001	−190.3 to −86.55

### Normative LV measures

Normative LV volumetric, strain and T1 mapping measures are provided in Table 1 as an average of a large group of mice (*n* = 46 for function assessment, 36 for T1 measurements). LV radial and circumferential strain mean values in WT mice were equal to 50.2 ± 10% and to −15.5 ± 2.8%, respectively. Table 2 shows the longitudinal distribution of radial and circumferential strains from the base to the apex of the LV. No significant difference was observed between basal, mid and apical slices in terms of radial strain (basal: 43 ± 13%, mid: 42 ± 14%, apical: 41 ± 17%; *p* = 0.96). Circumferential strain increased gradually from base to apex and was significantly higher at the apical level compared with basal or mid-LV levels (basal: −14 ± 1%, mid: −15 ± 2%, apical: −21 ± 3%; *p* < 0.0001). Radial and circumferential strain rate were equal to 25.2 ± 4.4 and to −7.8 ± 0.8 respectively.

**Table 2:** Radial and circumferential peak systolic strains from base to apex in the wild type group (*N* = 46).

	Basal	Mid	Apical	*p*-value
Radial peak systolic strain (%)	43 ± 13	42 ± 14	41 ± 17	0.96
Circumferential peak systolic strain (%)	−14 ± 1	−15 ± 2	−21 ± 3	< 0.0001

A notable difference in native T1 values was observed between the LV mid-ventricular and apical slices within the septal segment in WT mice. Additionally, native T1 ranged between 1380 ms and 1840 ms across LV segments, the highest values were seen in mid-lateral segments and in apical anterior segments. Post-contrast T1 ranged between 961 ms and 1149 ms across LV segments, the highest values were seen in mid-septal segments and in apical inferior segments.

### Myocardial strain and T1 mapping after MI

**Fig. 5:**
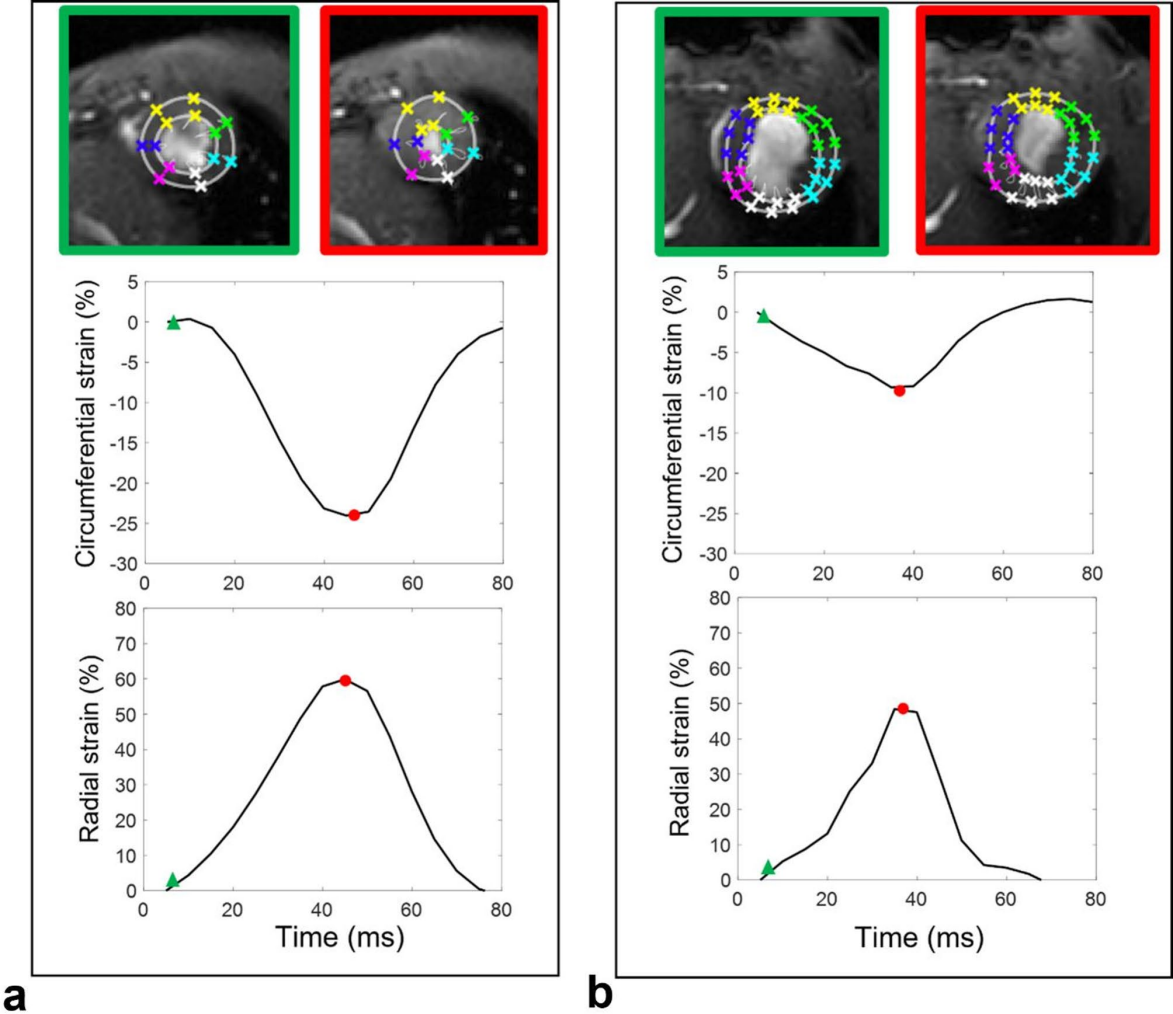
Examples of radial and circumferential strain curves in a wild type mouse (**a**) and in a mouse with an MI (**b**), which can be seen through a significant remodeling and thinning of the anterior wall on the end-systolic and end-diastolic images (top). Both circumferential and radial strain magnitudes were lower in the MI as compared to the wild type mouse.

**Fig. 6:**
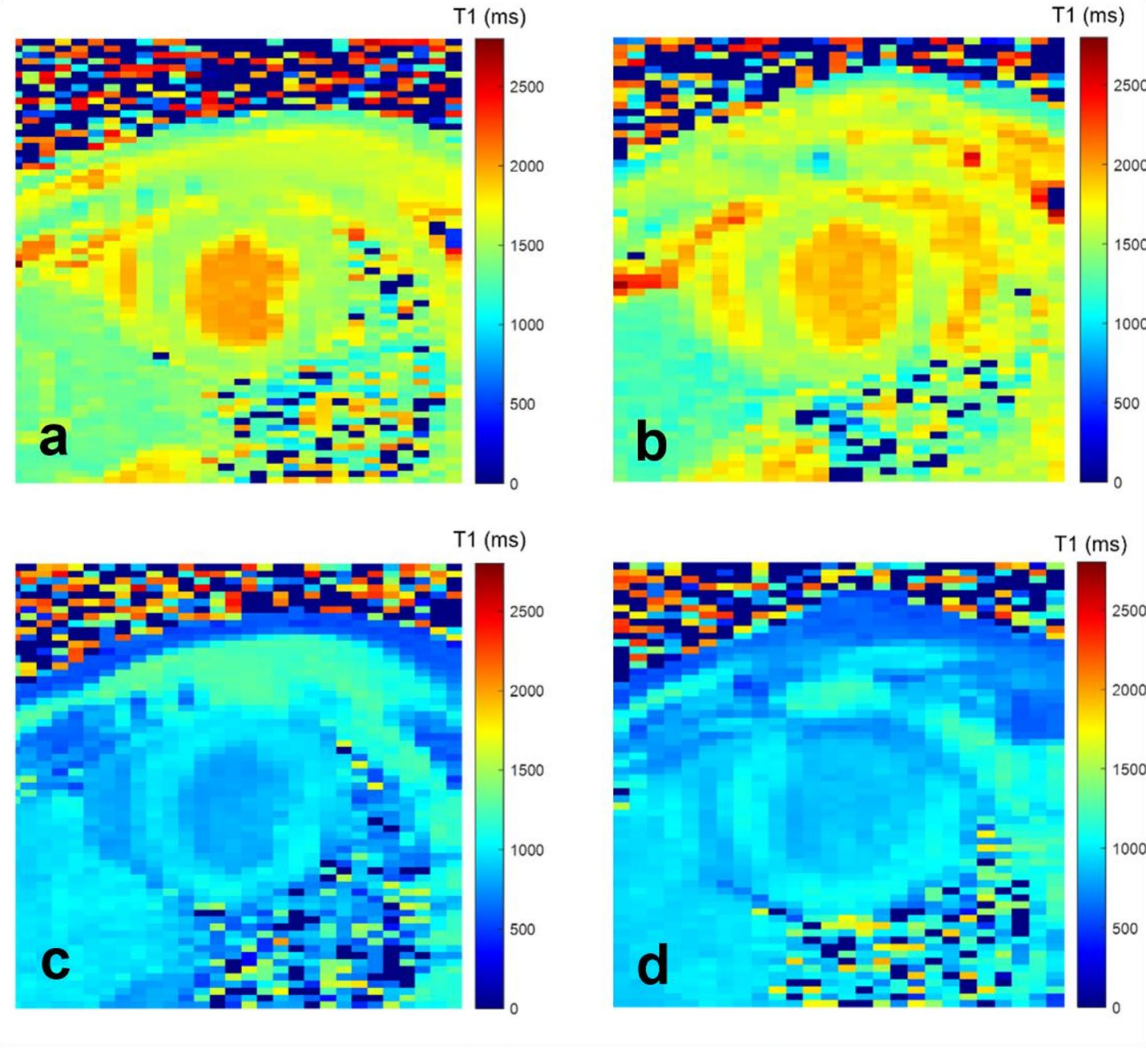
Examples of reconstructed T1 maps of a wild type mouse (left column, **a–c**) and a mouse with MI (right column, **b**–**d**) before (first row, **a**, **b**) and after (second row, **c**, **d**) contrast injection. A typical thinning of the anterior wall after the myocardial infarction is seen along with a local increase in native T1 value and decrease in post-contrast T1 value.

**Fig. 7:**
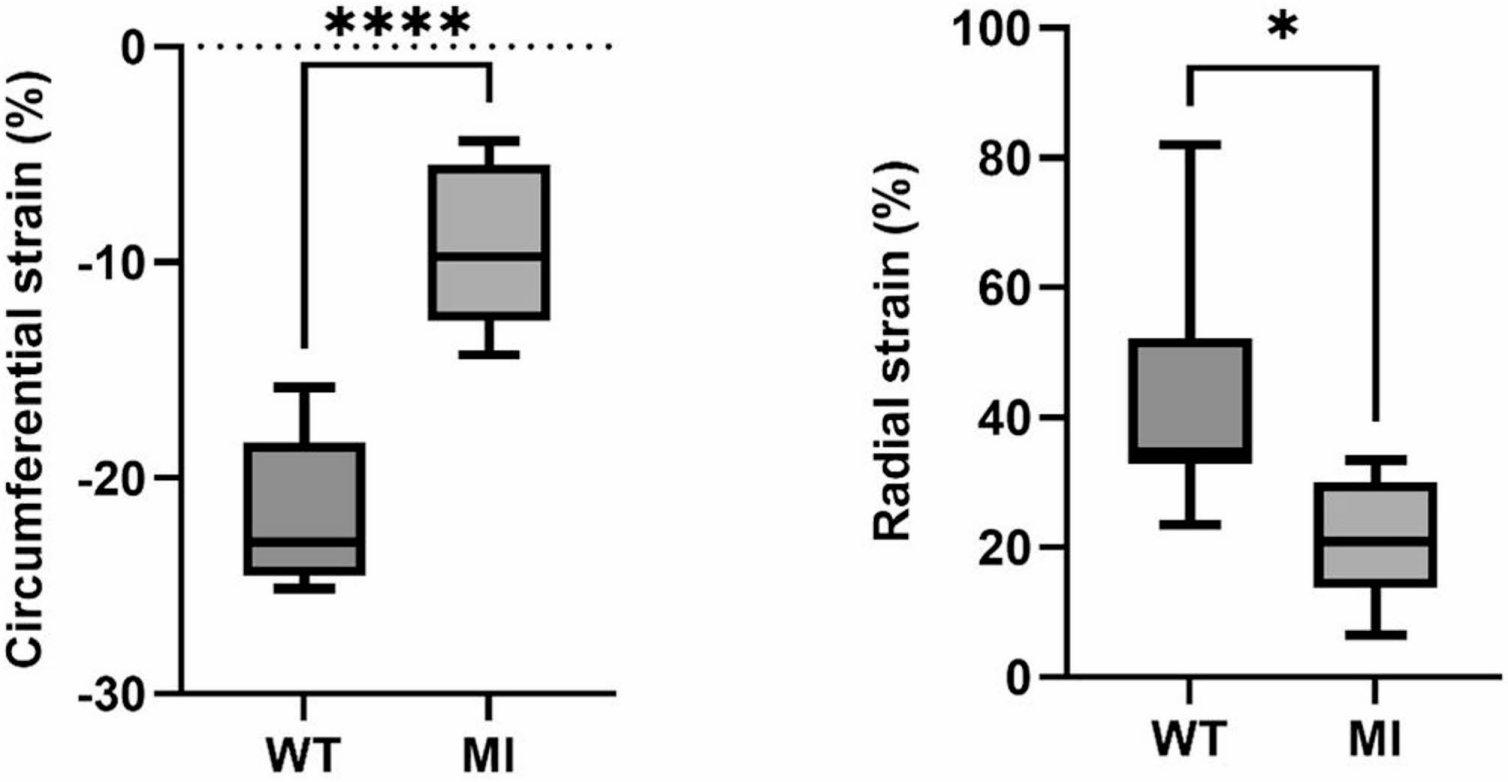
Circumferential and radial peak systolic strain at the LV apical level in the wild type (WT) and myocardial infarction (MI) groups.

Figures 5 and 6, respectively, illustrate myocardial circumferential and radial strain measures as well as native and post-contrast T1 maps in a WT mouse and in a mouse with MI indicating a drop in both circumferential and radial strain along with an elevation of native T1 and a decrease in post-contrast T1 in the anterior wall, corresponding to the MI region. Overall same drop in strain and post-contrast T1 as well as elevation in native T1 were found in the entire MI and WT groups (Table 1; Figs. 6 and 7).

Table 3 illustrates the change in strain between base to apex in the MI group. While basal strain showed a small compensatory elevation in the MI mice as compared to WT mice, there was a substantial drop in apical strain. Accordingly, strain differences between MI and WT mice were even larger when the analysis was restricted to the apical slice.

**Table 3:** Radial and circumferential peak systolic strains from base to apex in the myocardial infarction group (*n* = 23).

	Basal	Mid	Apical	*p*-value
Radial peak systolic strain (%)	53 ± 9	51 ± 20	21 ± 9	0.0048
Circumferential peak systolic strain (%)	−15 ± 4	−12.6 ± 3	−9 ± 3.8	0.07

Table 1 shows a trend of increase in native global T1 values after MI, but this did not reach statistical significance. Interestingly, the maximal native T1 value was significantly higher in the MI group as compared to the WT groups. Besides, both global and minimal post-contrast T1 values were significantly lower in the MI group as compared to the WT group.

**Fig. 8:**
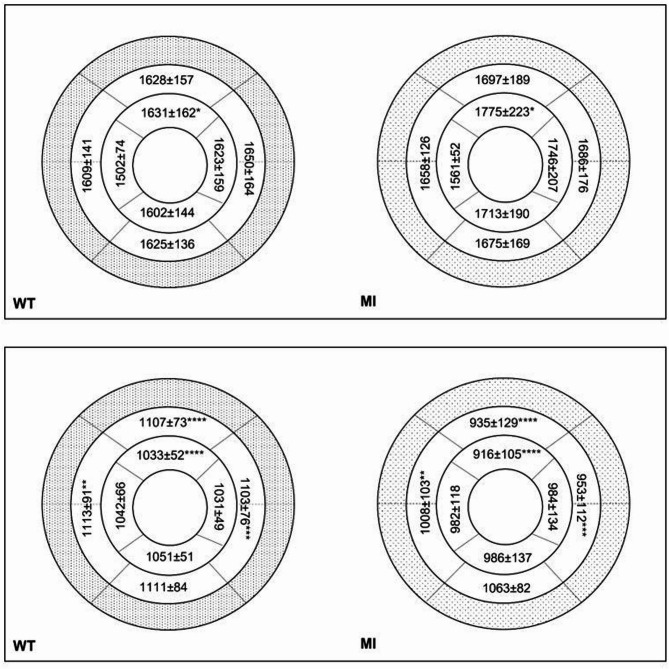
Bull’s eye representation of native (first row) and post-contrast (second row) T1 values in wild type (WT) mice and mice with myocardial infarction (MI). **: *p* < 0.01, ***: *p* < 0.001, ****: *p* < 0.0001.

Figure 8 illustrates native and post-contrast T1 values in a slice- and a wall- based analysis revealing higher native global T1 values, in the anterior wall of MI mice as compared to WT mice, reaching statistical significance on the apical slice. Post-contrast T1 also exhibited the lowest values in the anterior wall both on mid LV and apical slices and such values were significantly lower in MI mice as compared to the WT mice (*p* < 0.0001).

### Histology

Fibrosis quantification revealed a heterogeneous distribution, with values ranging from 13 to 50%, and a mean fibrotic area of 27 ± 15%. The results demonstrated a strong correlation between the histological degree of fibrosis and the mean and maximum native T1 values (R^2^ = 0,91, *p* = 0.04 and R^2^ = 0.89, *p* = 0.05, respectively), as well as the circumferential strain values (R^2^ = 0.77, *p* = 0.04), thereby supporting the validity of our imaging-based tissue characterization.

## Discussion

In this study, we have introduced an innovative acquisition protocol aimed at optimizing strain and T1 mapping measurements in small animals, in terms of acquisition and post-processing. To this end, we have customized a tracking software to suit the specific requirements of cardiac imaging in mice. These acquisition optimizations and software adaptations ensured a consistent analysis of strain and T1 mapping, thereby enhancing the overall quality and reliability of the results. The main findings of this study were established normative values in WT mice which were consistent with changes in MI model.

Our approach not only contributes to methodological advancements in preclinical cardiac imaging but also underscores the importance of tailored protocols and software solutions for overcoming challenges associated with small animal imaging.

Our sample size definition is two-fold: (1) we previously performed a study in human subjects, with the same strain software used here, including a scan rescan and sample size calculations for 5, 10 and 15% changes in strain values within all the main heart chambers including the left ventricle. Our previous study revealed that for a change in circumferential strain of 10%, we only need 11 samples^28^. Since 10% may be even lower than the drop in strain we expect to find in myocardial infarction, and since we expected to have more difficulties in estimating strain in a murine model than in human, we hypothesized that a sample size of 23 MIs would be widely sufficient to show the expected differences in strain. (2) such sample size has been then consolidated by literature findings in murine models: previous studies investigating MI in small animal models, particularly in assessing cardiac function and T1 mapping, have employed sample sizes comparable to those used in our study. For example, Elkenhans et al.^33^ conducted their analysis with 10 MI mice. Santer et al.^34^ included 8 MI mice, reporting a 23% overall mortality rate, highlighting the challenges of such experimental models. Räty et al.^35^ used 8 MI mice, confirming the feasibility of meaningful conclusions with this cohort size. Coolen et al.^36^ also relied on 8 MI mice, demonstrating that this sample size has been widely accepted in prior research.

Our software has been tailored to enable strain analysis for small animal MR images. For instance, post-processing adjustments were implemented to minimize flow artifacts, to customize the tracking algorithm to the small animal images size and resolutions and to adapt for possible trigger issues. These adaptations enhance the software’s capability to accurately assess strain in mouse, addressing specific challenges associated with spatial limitations, flow-related artifacts and ECG corruption in complex animal models and at high field. The refined post-processing techniques contribute to a more robust and precise analysis of strain, providing valuable insights into cardiac function in murine models.

The baseline circumferential and radial strain and strain rate in WT mice are in line with previous studies^34,37^ using MRI feature tracking or ultrasound speckle tracking. What sets up our study apart is the larger number of animals and the inclusion of T1 mapping in addition to strain analysis. WT strain computed in this study were slightly different than those of Gilson et al. and Suever et al.^38,39^ who used DENSE technique to measure strain. This difference could be explained by technical differences in measurement strategies focusing either on the deformation of myocardial borders or on the intra-myocardial deformation. Also, a special caution should be taken when comparing between small animal studies, as differences can be multifactorial and induced by differences in anesthesia, intrinsic strain of the mouse, heart rate, and temperature during the scan.

The increasing trend of circumferential strain from LV base to apex in healthy heart also supports consistency of our findings. Indeed, this phenomenon has been previously reported in humans^40,41^. Furthermore, the significant drop in circumferential and radial peak contractile strains after MI is consistent with prior studies. Such reduction of strain is associated with the increase of fibrosis synthesis and the decrease of collagen degradation^42^.

Although known as an accurate T1 measurement, inversion-recovery-based T1 mapping in the mouse heart is challenging, especially in infarcted hearts that feature poor ECG signal quality, which is why different approaches or acquisitions providing lower effective spatial resolution and, potentially, less accuracy have been used in the past. Nezafat et al.^43^ proposed a multi-shot 2D modified Look-Locker sequence for high-resolution T1 mapping in mice at a 3 T MRI clinical scanner. A further method for myocardial T1 mapping in mice has been proposed by Coolen et al.^36^ using a 3D intra-gate FLASH sequence in combination with a variable flip angle DESPOT1 (driven equilibrium single-pulse observation of T1) analysis. With this protocol, we aimed to evaluate a Look-Locker-based single-gradient echo sequence^27^ for its usefulness for studying myocardial infarction in the mouse. This sequence is more time-consuming than steady-state approaches, but, due to the single-echo acquisition per cardiac cycle, its spatial resolution is not blurred by cardiac motion. One drawback of the ECG-gated Look-Locker strategy, however, is its relative vulnerability in case of poor ECG quality that resulted in a significant number of discarded data sets in this study. Self-gated Look-Locker mapping techniques have been developed and successfully used recently to tackle these issues, however at the expense of longer measurement times^44^.

In the literature, there exists variability in both the dosage and type of contrast agent to be administered in mice. Different research endeavors employ diverse concentrations and formulations of contrast agents for imaging purposes, reflecting the absence of a standardized approach in preclinical investigations. The choice of contrast agent dosage in preclinical cardiac MRI varies significantly across studies, as reported in the literature. In their study, Chapon et al.^45^ administered a bolus of 0.6 mmol/kg of Gd-DTPA (Magnevist, Schering Healthcare, UK), a contrast agent commonly used in medical imaging. It is noteworthy that the dosage chosen for their experimental model was three times higher than the standard dose employed in human subjects. Bohl et al.^46^ employed an alternative contrast agent with a distinct dosage of 0.5 mmol/kg in their study. Other authors used gadodiamide (Omniscan, GE Healthcare, Oslo, Norway) as a contrast agent with a dose equivalent to 0.3 mmol/kg^47^. Similarly, Price et al.^48^ used 0.5 mmol/kg Gd-DTPA, and Yang et al.^49^ reported doses between 0.3 and 0.6 mmol/kg. More recently, Chaher et al.^50^ used a 0.8 mmol/kg dose of Gd, which aligns with our chosen contrast regimen in this study. This observation underscores the diversity in experimental approaches within the literature, highlighting the importance of considering such variations when interpreting and comparing results across different research studies.

Our choice of 0.8 mmol/kg was thoroughly optimized to obtain optimal myocardial nulling and ensure high-quality, interpretable images. This dose provided an acceptable contrast-to-noise ratio, facilitating reliable tissue characterization, while minimizing potential artifacts.

In our study, the changes of myocardial tissue characteristics secondary to fibrosis as depicted by T1 values were associated with subtle LV dysfunction that was detectable by circumferential strain. Furthermore, feature tracking showed high reproducibility in left ventricle global circumferential and longitudinal strain in healthy mice, whereas reproducibility of radial strain was limited^51^.

An essential aspect of MI-induced studies, including ours, involves considering the dominance pattern of coronary arteries and the precise level of ligature applied to the LAD in inducing myocardial infarction in mice^52,53^. The dominance of coronary arteries, whether left or right dominance, can influence the distribution and extent of ischemic injury. The specific location of ligature on the LAD is critical, as variations in ligation levels can lead to heterogeneous infarct sizes^54^ and affect the subsequent mapping results. Addressing these factors is crucial in the interpretation of our findings, as the dominance pattern and ligation site represent potential confounding variables that may influence the observed outcomes. By acknowledging these nuances, future work should aim to enhance the robustness and accuracy of our data interpretation, considering the impact of such confounders on the experimental results.

The first limitation of this study is that the histology analysis was only present in 5 mice. Indeed, the lack of histological examination in all mice restricts our ability to correlate the observed imaging changes in strain and T1 with underlying tissue characteristics. A comprehensive histological assessment would have provided valuable insights into the cellular and structural alterations after MI enhancing the interpretative depth of our findings. Future studies should consider incorporating histopathological analyses to bridge this gap and strengthen the overall understanding of the measured imaging manifestations.

Another limitation lies in the absence of pre-MRI ultrasound evaluation of the myocardial infarction. The lack of an initial ultrasound assessment prior to MRI introduces uncertainty regarding the exact nature and quality of the induced infarction. A pre-MRI ultrasound would have provided additional confirmation and a more comprehensive understanding of the baseline condition of the infarcted area, enabling a more accurate interpretation of the subsequent imaging data. An essential aspect of MI-induced studies, including ours, involves considering the dominance pattern of coronary arteries and the precise level of ligature applied to the LAD in inducing myocardial infarction in mice^52,53^. The dominance of coronary arteries, whether left or right dominance, can influence the distribution and extent of ischemic injury. The specific location of ligature on the LAD is critical, as variations in ligation levels can lead to heterogeneous infarct sizes^54^ and affect the subsequent mapping results. Addressing these factors is crucial in the interpretation of our findings, as the dominance pattern and ligation site represent potential confounding variables that may influence the observed outcomes.

A further limitation is that during the course of this study, the ECG monitoring system at our center was upgraded due to technical challenges encountered with the previous system. The small size of the mouse’s heart also posed a challenge, as ECG signal naturally exhibited low amplitude. After MI, this signal attenuation became more pronounced, making R-wave detection for gating particularly difficult. This issue was again perturbed by electromagnetic interference from the MRI environment, which can degrade ECG signal, especially in post-MI mice where the baseline signal is already weak. Irregular breathing patterns contributed to electrode instability, introducing additional noise into the signal. Magnetohydrodynamic effects^55,56^ also can introduce blurring artifacts. Considering these physiological and technical constraints, ECG-gated T1 mapping acquisitions in post-MI mice remain particularly challenging. However, despite these inherent limitations, our results remain consistent with prior studies in the field.

## Conclusion

Our proposed methodological innovation not only contributes to the refinement of existing imaging sequences but also holds promise for advancing our understanding of cardiac function and tissue characteristics using an adapted software. Myocardial interstitial fibrosis developed after MI can readily be detected with T1 mapping. In addition, cardiac MRI feature tracking shows a relationship between abnormal strain and abnormal LV tissue characteristics (native and post-contrast T1 mapping). These results may provide scientific basis for relevant basic and clinical research, particularly in the realm of drug therapy targeting increased fibrosis synthesis in patients after myocardial infarction.

## Data Availability

The datasets used and/or analysed during the current study are available from the corresponding author on reasonable request.

## Abbreviations

ANOVA: Analysis of Variance
CS: Circumferential Strain
DENSE: Displacement Encoding with Stimulated Echoes
DESPOT1: Driven Equilibrium Single Pulse Observation of T1
ECG: Electrocardiogram
ED: End diastole
EF: Ejection franction
ES: End systole
FLASH: Fast Low Angle Shot
FOV: Field-of-View
FT: Feature Tracking
LAD: Left Anterior Descending
LV: Left ventricle
MI: Myocardial Infarction
MRI: Magnetic Resonance Imaging
PARCC: Paris Cardiovascular Research Center
RS: Radial Strain
SENC: Strain Encoded
SNR: Signal-to-Noise Ratio
TI: Time to Inversion
WT: Wild type
